# Case Report: Rare Dynein Axonemal Heavy Chain 9 Mutations in a Han-Chinese Patient With Kartagener Syndrome

**DOI:** 10.3389/fmed.2022.893968

**Published:** 2022-06-13

**Authors:** Jingjing Feng, Junqing Li, Yong Du, Tianyun Shi, Lokesh Sharma, Zhijun Jie

**Affiliations:** ^1^Department of Pulmonary and Critical Care Medicine, Shanghai Fifth People’s Hospital, Center of Community-Based Health Research, Fudan University, Shanghai, China; ^2^Section of Pulmonary, Critical Care and Sleep Medicine, Yale University School of Medicine, New Haven, CT, United States

**Keywords:** kartagener syndrome, PCD, *DNAH9*, gene mutation, situs inversus

## Abstract

A 52-year-old woman presented with respiratory symptoms of productive cough and shortness of breath. She had suffered from repeated pneumonia. The CT scans revealed chronic sinusitis, tree bud signs in pulmonary imaging, and situs inversus. She received a primary diagnosis of Kartagener syndrome of primary ciliary dyskinesia (PCD) and a genetic examination was performed. Compound heterozygous mutations in dynein axonemal heavy chain 9 (*DNAH9*) were identified, which encoded outer dynein arms (ODAs) components. *DNAH9* mutations are relatively rare events in PCD, and this is the first report of PCD patients with *DNAH9* mutations in the Chinese population. Further, a literature review of mutations in PCD was conducted.

## Introduction

Kartagener syndrome is characterized by bronchiectasis, chronic sinusitis, and situs inversus, which is a subtype of primary ciliary dyskinesia (PCD) (1, 2). PCD is an autosomal recessive or X-linked genetic disorder in which the cilia are dysfunctional and fail to effectively transport secretions (3). The prevalence of PCD is about 1/16,000 to 1/20,000 (4). Kartagener syndrome is present in 40–50% of PCD cases. So far, around 40 cilia-related genes have been found to be associated with PCD (5, 6). Mutations in *DNAH9* are rarely reported. There is still no case reported in China. Herein we report a Kartagener syndrome case with *DNAH9* mutations in a Han-Chinese patient.

## Case Report

### Clinical Presentation

A 52-year-old female was admitted to the hospital due to recurrent cough, expectoration, and shortness of breath for more than 10 years. In her hometown, she frequently presented to the hospital with lung infections and was treated with antibiotics. She received a pulmonary function test in September 2021 in Shanghai, which indicated obstructive ventilatory dysfunction because FEV_1_/FVC was less than 70%. Consequently, she was diagnosed with chronic obstructive pulmonary disease (COPD). She was treated with long-acting beta2-receptor agonist (LABA) + long-acting cholinergic receptor antagonist (LAMA) (Olexin) inhalation for about 2 months. Two days prior to the current admission, she was presented with increased cough and yellow sputum. She had no fever, chills, fatigue, night sweats, progressive emaciation, chest pain, or other symptoms. She had a history of spontaneous pneumothorax because of ruptured bullae of the lung. However, she could not provide the X-ray and a detailed history of the previous therapies. Although clear that she had situs inversus, she was treated as a common pulmonary disease. She denied any history of PCD in any of her parents or risk factors such as inbreeding. She has a son and a daughter, both of them are healthy with no apparent signs of lung disease. There is no other person with similar symptoms in her family.

### Examination

On a physical examination, her blood pressure was 140/90 mmHg, pulse rate of 107 beats/min, respiratory rate of 15 times/min, and oxygen saturation of 98% at room air. On cardiovascular examination, heartbeats were audible on the right side of the chest. There were not any coarse crackles in both lungs. An initial investigation revealed a white cell count of 3,290/μL and a C reactive protein level of less than 1 mg/L. The arterial blood gas analysis showed her PO2 level was 111 mmHg when she received an oxygen flow rate of 3 L/min. There were not significant abnormal findings in liver and kidney functions, electrolytes, ESR, procalcitonin, peripheral blood lymphocyte subsets, markers of autoimmunity, and antinuclear antibody.

**FIGURE 1:**
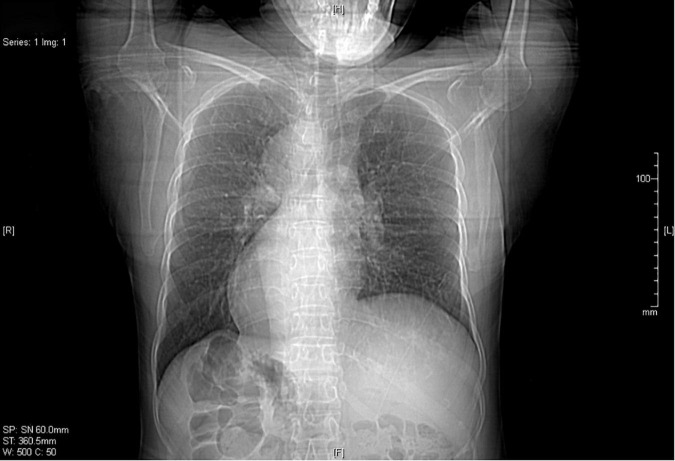
Chest X-ray shows dextrocardia in this patient with situs inversus.

**FIGURE 2:**
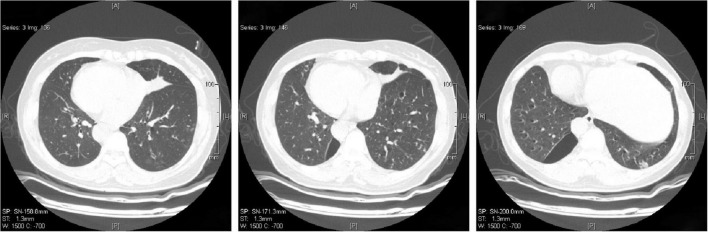
Chest computed tomography shows inflammation in both sides of lung lobes.

**FIGURE 3:**
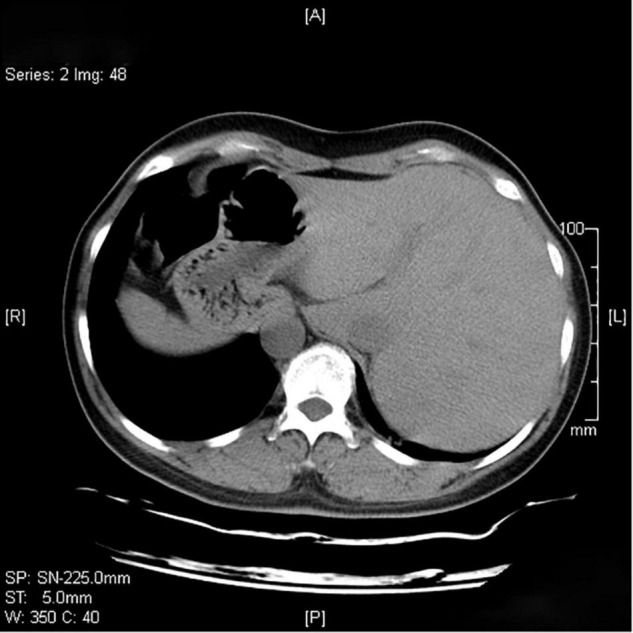
Computed tomography scan of the abdomen showing liver on the left.

**FIGURE 4:**
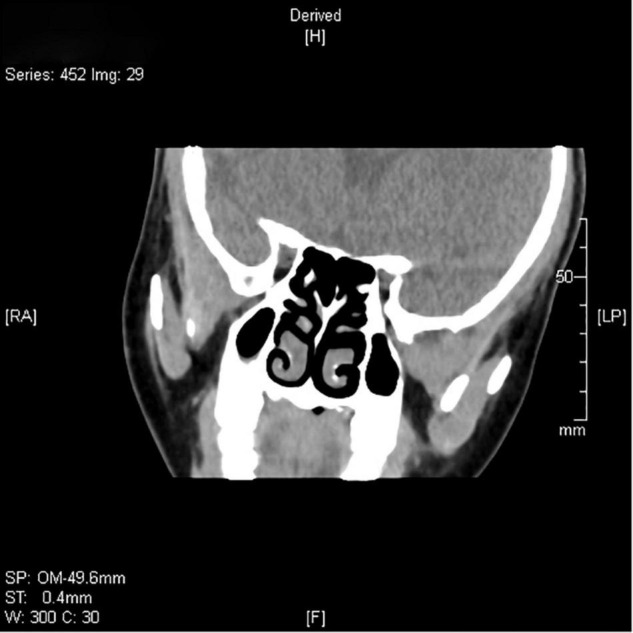
The paranasal sinus CT scan of the proband shows non-specific thickening of the mucosa on the bilateral maxillary sinuses.

**FIGURE 5:**
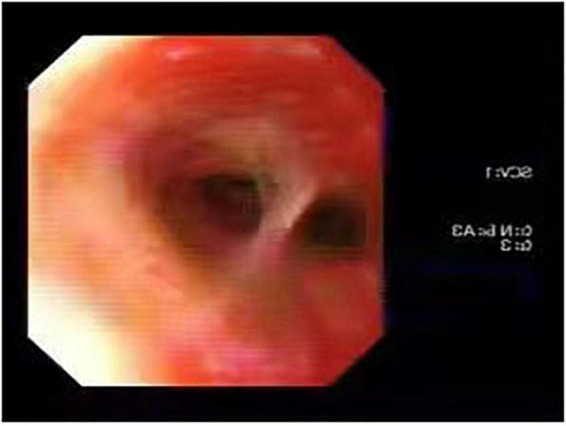
Fiberoptic bronchoscopy showed bronchial total inversion, bronchial mucosal inflammation in both lungs.

In sputum smear, Gram-positive cocci, suspected to be *Streptococcus* was identified. The bronchial alveolar lavage fluid (BALF) was obtained to perform metagenomic sequencing and *Nocardia gelsenkircheni* was identified. Chest X-ray (posteroanterior view) showed a cardiac shadow on the right side (Figure 1). Many tree-in-buds on both sides of lung lobes were found in Chest computed tomography (CT) (Figure 2). Abdominal CT confirmed complete situs inversus totalis (Figure 3). Otitis media tympanitis with effusion was found by an otolaryngologic examination (Figure 4). Fiberoptic bronchoscopy showed total bronchial inversion and bronchial mucosal inflammations in both lungs (Figure 5).

Given the history of repeated respiratory infections and situs inversus, combined with the physical examinations, she was suspected to have PCD. Following the European Respiratory Society (ERS) guidelines for the diagnosis of PCD (6), we intended to evaluate nasal nitric oxide (nNO) levels and perform a high-speed video analysis (HSVA). Unfortunately, we were unable to conduct those tests because of our technical limitations. We then performed genetic testing of her peripheral blood to confirm the diagnosis, according to the diagnostic criteria of the Chinese Expert Consensus on the Diagnosis and Treatment of Primary Ciliary Dyskinesia (7).

### Genome Sequencing

**FIGURE 6:**
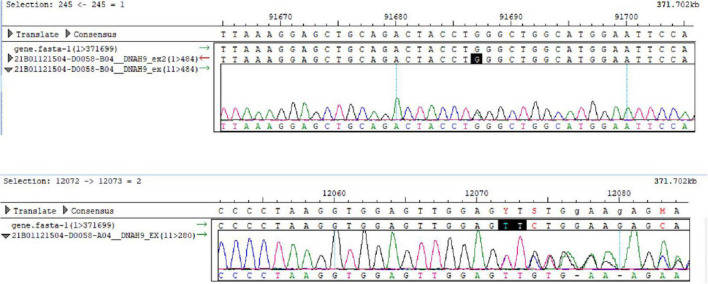
The results of Sanger sequencing in this case. Compound heterozygous mutations in the *DNAH9* gene are shown.

We performed whole-exome sequencing to detect the presence of any mutation(s). Two milliliters of peripheral venous blood preserved in an ethylenediamine tetraacetic acid (EDTA) coated tube was sent to the Shenzhen BGI Medical Test Laboratory. The sequencing was performed by capture high-throughput chip technology. Sanger sequencing was used to verify the mutations. The results showed that two suspected pathogenic mutations were located on chromosome 17; CHR17:11513858-11513859 and CHR17:11593470 (Figure 6), and detected in *DNAH9* gene associated with PCD type 40, which was partially related to the phenotype of the subjects with PCD. One of the mutations, *DNAH9* (NM_001372.3, c.760_761delTT, p.Phe254Leufs*6) is a frame-shift mutation caused by the deletion of two nucleotide TT at nucleotide positions 760-761 of the coding sequence of the gene, resulting in the phenylalanine codon at position 254 being changed to leucine, and a stop codon at position 260. The c.760_761delTT mutation is mentioned in ClinVar. Another mutation, *DNAH9* (NM_001372.3C, c.4331G > A, chr17:11593470) is a nonsense mutation caused by the substitution of nucleotide A with G, resulting in the leucine codon at position Trp being changed to a stop codon. We could not get blood samples of her parents or children for the gene analysis, but they had no clinical manifestation of PCD. This study was approved by Shanghai Ethical Review Board (2022-047).

### Clinical Management

Ampicillin tazobactam was administered to treat the infection, ambroxol hydrochloride was administered to reduce sputum, albuterol, and acetylcysteine were inhaled to dilate the airways and promote sputum excretion. After treatment, the symptoms were relieved, and she was discharged. The patient was then prescribed fordostine to eliminate phlegm and urmetrium bromide and verranterol for inhalation therapy.

## Discussion

Due to its low prevalence, Kartagener syndrome has been rarely reported, and *DNAH9* gene mutations are even rarer. In this study, we firstly reported *DNAH9* gene mutations in a Han-Chinese subject with Kartagener syndrome. *DNAH9* is involved in the function of motile node monocilia. The loss of *DNAH9* could impair ciliary motion and subsequently alter nodal flow, result in laterality defects (8). The loss-of function mutations in *DNAH9* cause motile cilia defects and situs inversus (9). *DNAH9* is a component of the type 2 ODA. The ODAs are responsible for the generation of ciliary motion. In the cilia of respiratory epithelium in mammals, there are at least two types of ODAs, the ODA type 1 containing *DNAH5* as well as *DNAH11* (10) and the ODA type 2 containing *DNAH5* and *DNAH9* (11). Loges et al reported eight mutations in the *DNAH9* gene among laterality defects and in patients with respiratory ciliary-beating defects (8). The mutated alleles of *DNAH9* which were previously reported are different from those presented in this study. Fassad et al analyzed three families of motile cilia defects and situs inversus, and also found five mutations in *DNAH9* genes (9). *DNAH9* gene encodes the heavy chain subunit of axonemal dynein, a large multi-subunit molecular motor. Axonemal dynein attaches to microtubules and hydrolyzes ATP to mediate the movement of cilia and flagella. The frequency of c.760_761delTT is 0.00004779 in GnomAD database and is mentioned in ClinVar. The frequency of c.4331G > A was not included in TOPMed or GnomAD database.

No case has been reported in domestic literature in China. It could also be due to a lack of genetic testing. Next generation gene sequencing and whole exome gene sequencing can not only diagnose PCD, but also find new mutations that lead to clinical manifestation of PCD. In the Chinese Expert Consensus on the Diagnosis and Treatment of Primary Ciliary Dyskinesia published in 2020, 12 literatures related to gene detection of PCD patients in China were summarized. A total of 17 cases of PCD with genetic mutations were reported, but no mutation in *DNAH9* gene was reported (7).

PCD manifests as a series of clinical presentations caused by ciliary motility disorders. Ciliary structural abnormalities in PCD patients include partial loss of ciliary motility arm, such as unilateral loss of lateral or medial ciliary motility arm, or complete loss of ciliary motility arm. The ciliary motility disorder caused by *DNAH9* gene mutation is mainly manifested as lack of ciliary tail motility, but not the total loss of ciliary motility. Loges et al reported that *DNAH9* mutations cause laterality defects with no or only mild respiratory symptoms such as recurrent airway infections instead of a classical respiratory PCD phenotype (8), which may be related to the patient’s mild clinical presentation. However, severe clinical manifestations in our case might be due to novel mutations in both the alleles in *DNAH9* gene.

In this case, respiratory symptoms were the initial manifestations of the patient, including repeated cough and sputum, accompanied by shortness of breath post physical activity, and imaging revealing total visceral inversion. What was inconsistent with Kartagener syndrome diagnosis was that the patient did not have typical bronchiectasis in lung imaging but had manifestations of diffuse bronchiolitis. Wang Lifei et al. (12) summarized the imaging characteristics of lungs and sinuses of 24 Kartagener syndrome patients. They found that Digital Radiography and CT mainly showed benign multiple small nodules, linear shadow and tree bud signs. The bronchiolitis changes were observed in 91.67% (22/24) patients, 58.33% (14/24) showed manifestations of diffuse panbronchiolitis (DPB). Finally, there were eight cases confirmed to be DPB. The patient’s sinus CT suggested the presence of mild paranasal sinusitis, which was consistent with the common manifestations of Kartagener syndrome. The patient suffered from spontaneous pneumothorax several years ago and was admitted to hospital in her hometown in rural China. The X-ray examination was performed during hospital stay and then she learned that she had situs inversus. However, her healthcare provider did not mention PCD or Kartagener syndrome to her due to a lack of awareness of this disease, which led to a delay in appropriate diagnosis.

The diagnosis of Kartagener syndrome can also be confirmed by electron microscopy examination of nasal mucosa or bronchial mucosa epithelium to observe the ultrastructure of cilia. In addition, nNO can assist the diagnosis. Due to technical limitations, electron microscopy and nNO were not performed in the patient.

As a type of PCD, Kartagener syndrome is an inherited disease, lacking definitive cure. It is primarily managed with symptomatic treatment, including infection prevention and control. Mucolytics are used to promote sputum excretion, and drugs promoting cilia movement can be administrated to improve host immunity to prevent infections.

Appropriate antibiotics can be used according to the bacterial culture and antibiotic sensitivity tests. In this case, multiple sputum smears showed Gram-positive cocci, suspected to be *Streptococcus*, and paracillin tazobactam was given to treat the infection. Ambroxol was administered to reduce phlegm and inhaled acetyl cysteine solution was given to facilitate sputum excretion.

The patient was treated with long-term inhalation of verranterol ummeronium bromide to dilate the airways to relieve shortness of breath and delay lung function decline.

Kartagener syndrome in patients with combined chronic rhinitis, sinusitis can be treated by local use of physiological saline, antibiotics and antibiotic containing nasal douches. Surgical excision is recommended for patients with long-term repeated respiratory infection symptoms, nasal secretions, presence of drug-resistant bacteria that affect quality of life.

Kartagener syndrome is a rare genetic disease, which is not difficult to diagnose. However, due to insufficient understanding of Kartagener syndrome by clinicians, misdiagnosis and missed diagnosis result in delayed treatment. As a genetic disease, it is very important to identify the mutation site, which can assist genetic counseling, including prenatal counseling. Genetic testing can be helpful to discover new pathogenic genes and deepen the understanding of the disease.

## Ethics Statement

The studies involving human participants were reviewed and approved by the Ethical Review Board. The authors declare that appropriate written informed consent was obtained for the publication of this manuscript and accompanying images. The patients/participants provided their written informed consent to participate in this study. Written informed consent was obtained from the individual(s) for the publication of any potentially identifiable images or data included in this article.

## Author Contributions

JF: preparation, funding acquisition, and writing—original draft. JL and LS: writing—review and editing. TS and YD: resources supply. ZJ: conceptualization and writing—review and editing. All authors contributed to the article and approved the submitted version.

## Conflict of Interest

The authors declare that the research was conducted in the absence of any commercial or financial relationships that could be construed as a potential conflict of interest.

## Publisher’s Note

All claims expressed in this article are solely those of the authors and do not necessarily represent those of their affiliated organizations, or those of the publisher, the editors and the reviewers. Any product that may be evaluated in this article, or claim that may be made by its manufacturer, is not guaranteed or endorsed by the publisher.
